# Safety data on in situ gelling bimatoprost loaded nanovesicular formulations

**DOI:** 10.1016/j.dib.2019.104361

**Published:** 2019-08-06

**Authors:** Monika Yadav, Ana Guzman-Aranguez, Maria J. Perez de Lara, Mandeep Singh, Joga Singh, Indu Pal Kaur

**Affiliations:** a University Institute of Pharmaceutical Sciences, Panjab University, Chandigarh, India; b Faculty of Optics and Optometry, Department of Biochemistry and Molecular Biology, Universidad Complutense de Madrid, Madrid, Spain

**Keywords:** Cytotoxicity, Ocular toxicity, In situ gelling, Acute and repeat dose toxicity, Sub-conjunctival implants, Cell lines

## Abstract

In vitro cytotoxicity and in vivo acute and 7 days repeat-dose ocular toxicity studies, were conducted in rabbits, in accordance with the Organisation for Economic Co-operation and Development (OECD) guidelines, for bimatoprost loaded nanovesicular aqueous dispersion (BMT-NV) and its in-situ gelling sub-conjunctival implant (BMT-NV-IM). For details on the preparation and evaluation of BMT-NV and its BMT-NV-IM for the control of glaucoma, please refer to ‘Bimatoprost loaded nanovesicular long-acting sub-conjunctival in-situ gelling implant: In vitro and in vivo evaluation’ (Yadav et al., 2019). The in vivo ocular toxicity was performed only after confirming dermal safety, as required by OECD. Histological evaluation of various ocular tissues, following sub-conjunctival implantation with BMT-NV-IM, was done for ocular tolerance studies.

**Table:** Specifications Table

Subject area	Pharmacology, Toxicology and Pharmaceutical Sciences
More specific subject area	Nanotechnology
Type of data	Table Image Text file
How data was acquired	Plate reader (BioTek, Winooski, VT, USA), morphological examination and microscopy
Data format	Raw, Analyzed
Experimental factors	Cell proliferation cytotoxicity (MTT assay) studies were conducted in vitro using various cell lines. In vivo acute and 7 days repeat-dose ocular toxicity studies were conducted in rabbits, in accordance with the OECD guidelines after instillation of bimatoprost loaded nanovesicular aqueous dispersion (BMT-NV) and its in-situ gelling sub-conjunctival implant (BMT-NV-IM).
Experimental features	% Viability of stratified HCLE (human corneal - limbal epithelial), HCjE (human conjunctival epithelial) and R28 (retinal neuronal) cells was determined, after exposure to various formulations, taking viability of untreated control cells as 100%. For in vivo toxicity studies treated rabbit eyes were observed for clinical signs of irritation, inflammation, or infection, for the suggested period of study. Safety was also confirmed by observing histological structure and integrity of the rat eye, at 2 days, 1 week, 1 month and 2-month post administration of the formulations.
Data source location	Nanotechnology lab, University Institute of Pharmaceutical Sciences, Panjab University, Chandigarh, India
Data accessibility	Data is with this article
Related research article	**Author's name**: Monika Yadav, Ana Guzman-Aranguez, Maria J Perez de Lara, Mandeep Singh, Joga Singh, Indu Pal Kaur **Title:** Bimatoprost loaded nanovesicular long-acting sub-conjunctival in-situ gelling implant: In vitro and in vivo evaluation. **Journal:** Materials Science and Engineering: C **DOI:** doi.org/10.1016/j.msec.2019.05.015

**Table:** 

**Value of the data** • Work describes two-tier safety evaluation of novel nanovesicular ocular drops and a conjunctival in situ-gelling implant of bimatoprost for use in control of glaucoma. • In vitro cytotoxicity studies conducted in human corneal limbal epithelial (HCLE), human conjunctival epithelial (HCjE), and human retinal (R28) cell lines at two to three doses indicated that encapsulation of BMT into nanovesicular system was safe and could improve the safety over marketed BMT drops. • In vivo dermal toxicity studies (preamble to ocular toxicity) and acute and repeat dose ocular toxicity studies as per OECD guidelines also established complete safety of the developed formulation

## 1 Data

### 1.1 BMT loaded nanovesicles (BMT-NVs) and their incorporation into a gel (BMT-NV-GEL)

BMT-NVs and BMT-NV-GEL was prepared as discussed in the main article [1].

### 1.2 Cytotoxicity studies

All ingredients employed for preparing the BMT-NV-GEL were of biodegradable and biocompatible nature and were indicated to be safe for ocular use at the employed concentration. Poloxamer 407 (P407) and carbopol 934P have been already used in FDA approved ophthalmic formulations [2].

**Fig. 1:**
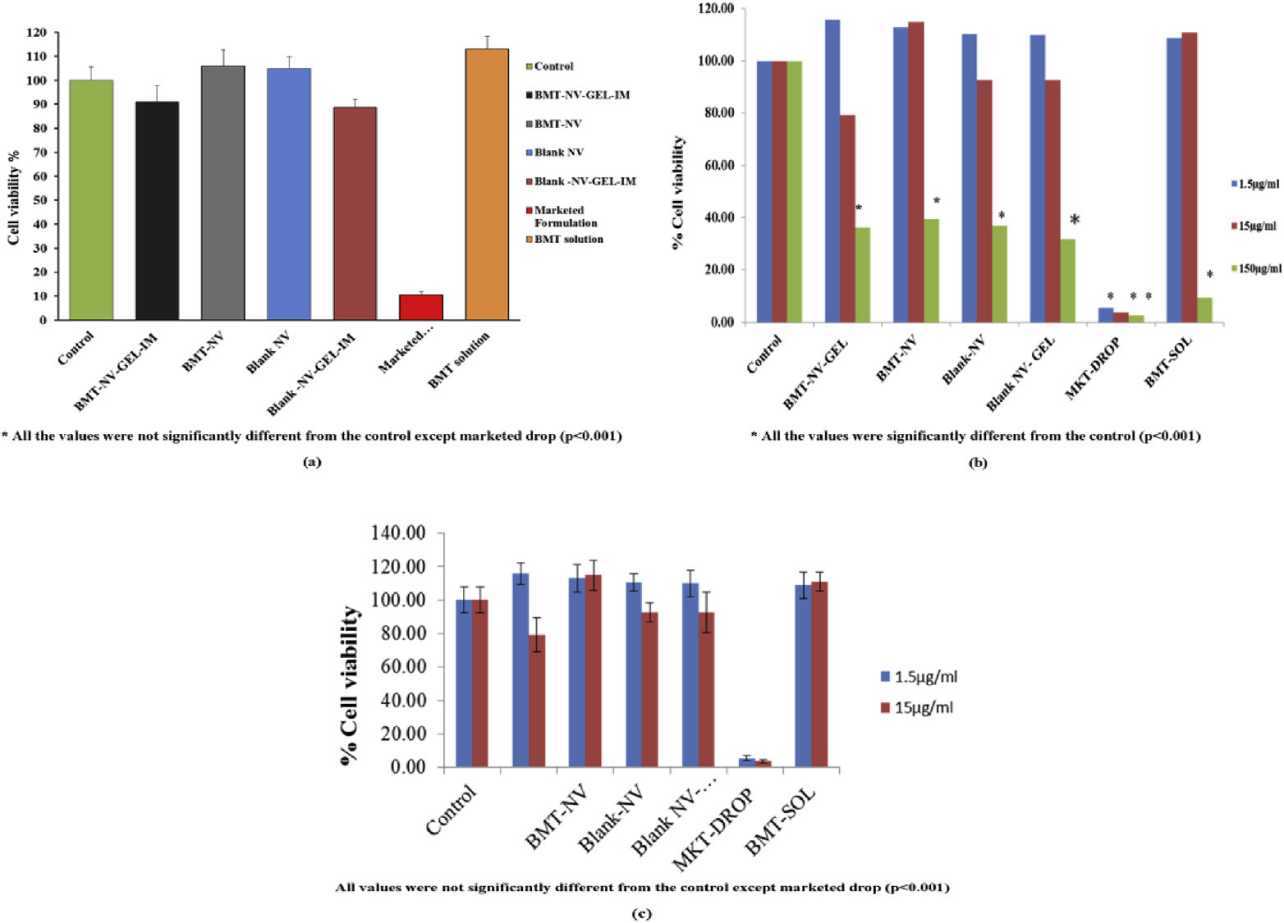
% cell viability in HCLE (a), HCjE (b) and R28 (c) cells at varying concentration after incubation for 24 h. All values are similar (p < 0.001) except those marked^∗^.

Both BMT-NV and BMT-NV-GEL were safe and did not show any statistically significant (p < 0.001) cytotoxicity (Fig. 1, Tables S1–S6 (raw data for Fig. 1, added as supplementary data)) when administered to HCLE (150 μg/ml), HCjE (1.5, 15) and R28 (1.5, and 15 μg/ml) cell lines for 24 h. Since BMT-NV-GEL is to be used as in-situ gelling ocular drops as well as subconjunctival implant so both HCLE and HCjE cell lines were used. However exposure to high concentration of 150 μg/ml in case of conjunctival cell lines HCjE showed significant cytotoxicity. It may be noted that we expect a slow release from the subconjunctival area, and at no time is the concentration expected to reach as high as 150 μg/ml; two lower doses i.e 1.5 and 15 μg/ml show complete protection. It may also be noted that exposure of HCjE cells to 150 μg/ml of free BMT is also toxic (∼10% viability; Table S4). In comparison BMT NV and BMT NV gel are 4 times less toxic (∼40% viability; Table S4).

Lower concentrations were also used for R28 cell lines which showed no cytotoxicity at 1.5 and 15 μg/ml.

Marketed drops (Lumigan drops) showed significant cell death in all three cell lines at all concentrations, and this result is not surprising as it has been published by other researchers [3], [4], [5], [6] that benzalkonium chloride (BAK), the preservative present in the marketed formulation can induce cell death in in vitro experiments.

### 1.3 In vivo safety studies in rabbits

OECD 405 recommends that in vivo eye irritation/corrosion test, should be conducted after the in vivo dermal safety (OECD testing guideline 404) of a product/substance is confirmed (AEI, 2002) [7].

The score for both the (i) acute dermal irritation/corrosion study (compiled in Table 1); and the (ii) acute eye irritation/corrosion (compiled in Table 2) was zero. This clearly demonstrates a non-irritant/corrosive nature of BMT-NV-GEL when applied to dermal and ocular tissues (topical instillation), and hence are concluded to be safe for ocular use.

**Table 1:** Acute dermal irritation/corrosion study of BMT-NV-GEL in rabbits.

Skin reactions	Time in h															Total count
	Animal 1					Animal 2					Animal 3					
	0	1	24	48	72	0	1	24	48	72	0	1	24	48	72	
Erythema	0	0	0	0	0	0	0	0	0	0	0	0	0	0	0	0/40
Oedema	0	0	0	0	0	0	0	0	0	0	0	0	0	0	0	0/40
Final total score																0/80

(Scoring 0–4 was done as described in OECD guidelines 404, as per Table 1: Grading of skin reactions, page 7 of [7]).

**Table 2:** Single instillation acute eye irritation/corrosion study of BMT-NV-GEL in rabbit eye.

Tissue of the eye	Time in h															Total count
	Animal 1					Animal 2					Animal 3					
	0	1	24	48	72	0	1	24	48	72	0	1	24	48	72	
Cornea	0	0	0	0	0	0	0	0	0	0	0	0	0	0	0	0/60
Iris	0	0	0	0	0	0	0	0	0	0	0	0	0	0	0	0/30
Conjunctiva	0	0	0	0	0	0	0	0	0	0	0	0	0	0	0	0/45
Chemosis	0	0	0	0	0	0	0	0	0	0	0	0	0	0	0	0/60
Grand Total score																**0/195**

(Scoring was done as defined in OECD guidelines 405 as per Table 1: Grading of ocular lesions (page: 8 of [9]).

Repeated instillation acute study was performed in view of the fact that glaucoma requires life long treatment and will need frequent instillation of developed formulation for effective control of IOP. Similar studies have been reported by us earlier [8]. The scores obtained from this study (Table 4) also prove the system to be safe for repetitive ocular use.

Similarly aggressive therapy viz. chronic repeat instillations (5 times at 5 minute interval) for a period of one week was also evaluated and BMT-NV-GEL was still found to be safe (Table 3).

**Table 3:** Repeat instillation (five times a day) acute eye irritation/corrosion study of BMT-NV-GEL in rabbit eye.

Ocular tissue	Scores of rabbit 1					Scores of rabbit 2					Scores of rabbit 3					Score
	0 h	1 h	24h	48h	72h	0 h	1 h	24h	48h	72h	0 h	1 h	24h	48h	72h	
Cornea	0	0	0	0	0	0	0	0	0	0	0	0	0	0	0	0/60
Iris	0	0	0	0	0	0	0	0	0	0	0	0	0	0	0	0/30
Conjunctiva	0	0	0	0	0	0	0	0	0	0	0	0	0	0	0	0/45
Chemosis	0	0	0	0	0	0	0	0	0	0	0	0	0	0	0	0/60
Total score																**0/195**

Scoring was done as described under Table 2.

**Table 4:** Scores of chronic repeat instillation irritation/corrosion study on BMT-NV-GEL.

Ocular tissue		Cornea	Iris	Conjunctiva	Chemosis	Score	Total Score
rabbit 1	0h	0	0	0	0	0/104	0/312
	1d	0	0	0	0		
	2d	0	0	0	0		
	3d	0	0	0	0		
	5d	0	0	0	0		
	6d	0	0	0	0		
	7d	0	0	0	0		
rabbit 2	0h	0	0	0	0	0/104	
	1d	0	0	0	0		
	2d	0	0	0	0		
	3d	0	0	0	0		
	5d	0	0	0	0		
	6d	0	0	0	0		
	7d	0	0	0	0		
rabbit 3	0h	0	0	0	0	0/104	
	1d	0	0	0	0		
	2d	0	0	0	0		
	3d	0	0	0	0		
	5d	0	0	0	0		
	6d	0	0	0	0		
	7d	0	0	0	0		

Scoring was done as described under Table 2.

### 1.4 Ocular tolerance evaluation

**Fig. 2:**
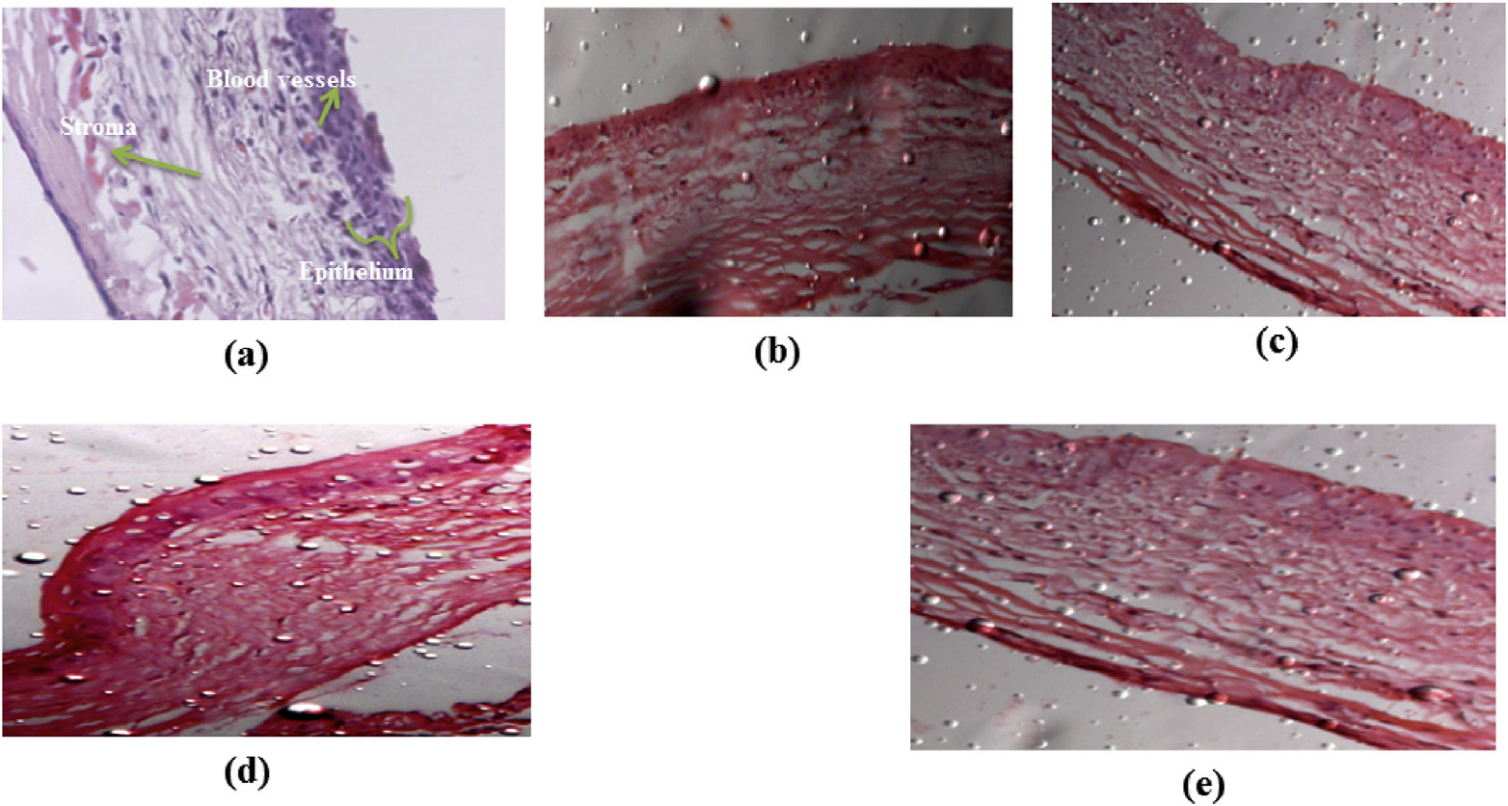
Optical microscopic pictures showing histological section of (a) naïve conjunctival tissue and conjunctival tissue of eyes post-treatment (b) 2 days, (c) 1 week, (d) 1 month, and (e) 2 month indicating absence of any untoward reactions.

Subconjunctival injection of BMT-NV-GEL implant [8] resulted in the formation of a bleb and conjunctival hyperemia (mild and transient) in 2 out of 4 injected eyes, which was resorbed completely within 48 h after injection. All eyes appeared normal and similar to the pre-injected or uninjected eyes after 48 h. The rats tolerated the procedure well, showing no sign of distress or pain during or immediately following administration. There were no signs of any irritation, swelling or redness and infection in any of the injected eye during the study. At the end of the study period, the residual gel was no longer observed and it is assumed that the gel dissolved completely. Particular attention was directed toward the sclera and conjunctival tissue surrounding the injection site during histological examination. The tissue appeared normal and we did not detect any signs of inflammation such as accumulation of macrophages, lymphocytic infiltrate, or evidence of giant cells (Fig. 2).

## 2 Experimental design, materials and methods

### 2.1 Establishing safety of the developed system

#### 2.1.1 Cytotoxicity studies

Viability of stratified HCLE (human corneal - limbal epithelial), HCjE (human conjunctival epithelial) and R28 (retinal neuronal) cells was measured by the cell proliferation assay using MTT (3-(4,5-Dimethyl-2-thiazolyl)-2,5-diphenyl-2H-tetrazolium bromide). Cells were grown in keratinocyte serum-free medium (Invitrogen, Carlsbad, CA) maintained at 37 °C in 5% CO_2,_ and supplemented with 25 μg/ml bovine pituitary extract, 0.4 mM CaCl_2,_ 0.2 ng/mL epidermal growth factor (EGF) and other suitable antibiotics. For suitable stratification and differentiation, the serum-free Invitrogen medium was replaced (at confluence) with Dulbecco's minimal essential medium (DMEM)/F12 medium, supplemented with 10% calf serum and 10 ng/ml EGF for a period of 7 days [10]. R28 cells were grown in DMEM (Invitrogen, Carlsbad, CA) supplemented with fetal bovine serum (10%), glutamine (2 mM), gentamicin (0.21 mM), non-essential amino acids and MEM vitamins (1% each).

HCLE, HCjE and R28 cells were exposed, after stratification in DMEM/F12 medium, to various test samples viz. BMT-NV, Blank-NV, BMT-NV-GEL, Blank-NV-GEL, BMT- SOL and marketed formulation (Lumigan® 0.03% drops) for 24h. MTT assay was then used to determine cell viability [4]. Fresh MTT solution (0.5 mg/ml) was added to variously exposed cells and incubated for 2 h at 37 °C. The purple formazan complex released from the cells after lysis was then dissolved in dimethyl sulfoxide. Absorbance of these samples was read on Gen 5 plate reader (BioTek, Winooski, VT, USA) at 570 nm. The reading was corrected for background by subtracting the absorbance measured at 690 nm from that at 570 nm. The mean absorbance value of untreated cells was taken as 100% viability and all results are expressed as % cell viability compared to these cells. All experiments were repeated three times (n = 3).

#### 2.1.2 BMT-NV-GEL in vivo safety studies as ocular drops

Safety assessment for ocular application was approved by the Institutional Animals Ethics Committee, Panjab University, Chandigarh, India (PU/IAEC/S/16/110, dated11/7/2016) and performed as per the OECD guidelines.

#### 2.1.3 Dermal irritation/corrosion test as per OECD guideline 404 (ADI, 2002) [7]

It is required to perform this test prior to the ocular irritation test. Only those test substances, which are safe for dermal use, are further instilled in to the rabbit eye for eye irritation test [7].

Six-month-old female albino rabbits, weighing between 1.3 and 1.7 kg, and with intact skin were used. The dorsal trunk area of rabbits was shaved using hair clippers and a depilatory cream, 1 day prior to the test. To 6-cm^2^ gauze, 0.5 mg of BMT-NV-GEL was applied uniformly. The gauze was fixed for 4h on the shaved skin with a non-irritating tape, ensuring complete contact with the skin surface. Care was taken to apply the gauze on a site away from easy access of the animal by mouth or by limbs and to ensure that animal may not ingest the patch. The uncovered shaved area was taken as control. After 4h of application, the gauze piece was removed, and the site was examined after 1h for any signs of erythema, oedema or redness. The test was initially performed on one rabbit and only proceeded with the other two animals if no reaction was observed on the first animal.

#### 2.1.4 Eye irritation/corrosion test as per OECD guideline 405 (AEI, 2002) [9]

Lower lid of right eye of each rabbit (n = 3) was pulled to create a space in the conjunctival sac and BMT-NV-GEL (0.1 ml) was administered either once or five X 0.1 ml instillations were made at 5 min intervals (for repeat test). Contralateral left eye was taken as the control for each animal. After instillation, each eye was examined at an interval of 1, 24, 48, and 72 h and scored for any reactions as described in the OECD guidelines (Table: 1, page: 8, reference: [9]). A chronic repeat dose study included administration of BMT-NV-GEL (0.1 ml), five X 0.1 ml instillations at 5 min intervals, every day for 7 days, to confirm safety of the formulation for long term therapy.

#### 2.1.5 Ocular tolerance evaluation

The effect of BMT-NV-GEL implant on structure and integrity of the administered eye (left) was determined at 2 days, 1week, 1 month and 2-month post administration. Right eye of all the treated animals was taken as control. Both the eyeballs were incised after sacrificing the animals and washed with saline. Then they were fixed in 8% w/w formalin solution and dehydrated in an alcohol gradient. After this the eyeballs were put in melted paraffin, which was then solidified to form a block. Cross-sections (<5 μm) cut from the latter, were observed microscopically (Nikon eclipse 90i, Japan), after haematoxyline and eosine (H and E) staining, for any pathological alterations [11].
