# Supplementary material for: Safety data on in situ gelling bimatoprost loaded nanovesicular formulations
Source: Data Brief. 2019 Aug 6;25:104361. doi: 10.1016/j.dib.2019.104361 (PMC6715818; doi:10.1016/j.dib.2019.104361)
Supplement: Supplementary file 1 [file mmc1.docx]

**Table S1: % cell viability in HCLE cell lines (Raw data of figure 1 A)**

| **Groups** | **I** | **II** | **III** | **Mean** | **% Viability** |
| --- | --- | --- | --- | --- | --- |
| **Control** | 0.708 | 0.639 | 0.651 | 0.666 | 100.00 |
| **BMT NV gel** | 0.649 | 0.56 | 0.610 | 0.606 | 91.04 |
| **BMT NV** | 0.706 | 0.751 | 0.660 | 0.705 | 105.96 |
| **Blank NV** | 0.671 | 0.699 | 0.727 | 0.699 | 104.97 |
| **Blank NV gel** | 0.580 | 0.576 | 0.616 | 0.590 | 88.69 |
| **Market Formulation** | 0.062 | 0.074 | 0.076 | 0.070 | 10.65 |
| **BMT solution** | 0.714 | 0.757 | 0.786 | 0.752 | 113.00 |

**Table S2: % cell viability in HCjE cells after exposure to 1.5 µg/ml dose (Raw data at dose of 1.5 µg/ml of figure 1B)**

| **Groups** | **Dose**  **1.5 µg/ml** | **I** | **II** | **III** | **Mean** | **% Viability** |
| --- | --- | --- | --- | --- | --- | --- |
| **Control** | | 0.393 | 0.337 | 0.366 | 0.365 | 100.00 |
| **BMT NV gel** | | 0.411 | 0.451 | 0.407 | 0.423 | 115.80 |
| **BMT NV** | | 0.377 | 0.400 | 0.460 | 0.412 | 112.86 |
| **Blank NV** | | 0.394 | 0.424 | 0.391 | 0.403 | 110.38 |
| **Blank NV gel** | | 0.372 | 0.410 | 0.422 | 0.401 | 109.85 |
| **Market Formulation** | | 0.015 | 0.018 | 0.025 | 0.019 | 5.38 |
| **BMT solution** | | 0.429 | 0.392 | 0.371 | 0.397 | 108.76 |

**Table S3: % cell viability in HCjE cells after exposure to 15µg/ml dose (Raw data at dose of 15 µg/ml of figure 1B)**

| **Groups** | **Dose**  **15 µg/ml** | **I** | **II** | **III** | **Mean** | **% Viability** |
| --- | --- | --- | --- | --- | --- | --- |
| **Control** | | 0.401 | 0.395 | 0.347 | 0.381 | 100.00 |
| **BMT NV gel** | | 0.258 | 0.326 | 0.321 | 0.301 | 79.26 |
| **BMT NV** | | 0.405 | 0.473 | 0.435 | 0.437 | 114.87 |
| **Blank NV** | | 0.328 | 0.367 | 0.363 | 0.352 | 92.56 |
| **Blank NV gel** | | 0.381 | 0.379 | 0.299 | 0.353 | 92.65 |
| **Market Formulation** | | 0.013 | 0.015 | 0.014 | 0.014 | 3.67 |
| **BMT solution** | | 0.442 | 0.421 | 0.404 | 0.422 | 110.87 |

**Table S4: % cell viability in HCjE cells after exposure to 150 µg/ml dose (Raw data of figure 1B at 150 µg/ml)**

| **Groups** | **Dose**  **150 µg/ml** | **I** | **II** | **III** | **Mean** | **% Viability** |
| --- | --- | --- | --- | --- | --- | --- |
| **Control** | | 0.482 | 0.453 | 0.424 | 0.453 | 100.00 |
| **BMT NV gel** | | 0.139 | 0.204 | 0.150 | 0.164 | 36.28 |
| **BMT NV** | | 0.160 | 0.182 | 0.195 | 0.179 | 39.51 |
| **Blank NV** | | 0.117 | 0.191 | 0.192 | 0.167 | 36.89 |
| **Blank NV gel** | | 0.120 | 0.197 | 0.114 | 0.144 | 31.81 |
| **Market Formulation** | | 0.017 | 0.009 | 0.007 | 0.011 | 2.49 |
| **BMT solution** | | 0.035 | 0.048 | 0.044 | 0.042 | 9.38 |

**Table S5: % cell viability in R28 cells after exposure to 1.5 µg/ml dose (Raw data at 1.5 µg/ml of figure 1 C)**

| **Groups** | **Dose**  **1.5 µg/ml** | **I** | **II** | **III** | **Mean** | **% Viability** |
| --- | --- | --- | --- | --- | --- | --- |
| **Control** | | 0.475 | 0.407 | 0.442 | 0.442 | 100.00 |
| **BMT NV gel** | | 0.524 | 0.531 | 0.479 | 0.511 | 115.79 |
| **BMT NV** | | 0.486 | 0.470 | 0.540 | 0.498 | 112.86 |
| **Blank NV** | | 0.476 | 0.514 | 0.472 | 0.487 | 110.37 |
| **Blank NV gel** | | 0.445 | 0.500 | 0.511 | 0.485 | 109.85 |
| **Market Formulation** | | 0.017 | 0.022 | 0.030 | 0.023 | 5.376 |
| **BMT solution** | | 0.519 | 0.474 | 0.448 | 0.480 | 108.75 |

**Table S6: % cell viability in R28 cells after exposure to 15µg/ml dose (Raw data at 15 µg/ml of figure 1 C)**

| **Groups** | **Dose**  **15 µg/ml** | **I** | **II** | **III** | **Mean** | **% Viability** |
| --- | --- | --- | --- | --- | --- | --- |
| **Control** | | 0.393 | 0.387 | 0.340 | 0.373 | 100.00 |
| **BMT NV gel** | | 0.254 | 0.329 | 0.303 | 0.295 | 79.26 |
| **BMT NV** | | 0.396 | 0.463 | 0.426 | 0.428 | 114.87 |
| **Blank NV** | | 0.321 | 0.359 | 0.355 | 0.345 | 92.56 |
| **Blank NV gel** | | 0.371 | 0.372 | 0.293 | 0.345 | 92.64 |
| **Market Formulation** | | 0.010 | 0.017 | 0.012 | 0.013 | 3.66 |
| **BMT solution** | | 0.433 | 0.417 | 0.390 | 0.413 | 110.87 |
